# PickAMoo: LIDAR-enhanced mask R-CNN segmentation for precision weight estimation in dairy cattle using smartphone imaging

**DOI:** 10.1038/s41598-026-54742-3

**Published:** 2026-05-23

**Authors:** Oleksiy Guzhva, Emma Ternman, Mikaela Lindberg, Evgenij Telezhenko, Cecilia Kronqvist

**Affiliations:** 1 Department of Biosystems and Technology, Swedish University of Agricultural Sciences, Box 103, 230 53 Lomma, Sweden; 2 Department of Applied Animal Science and Welfare, Swedish University of Agricultural Sciences, Box 7024, 750 07 Uppsala, Sweden; 3 Faculty of Biosciences and Aquaculture, Nord University, Box 2501, 7729 Steinkjer, Norway

**Keywords:** Computational biology and bioinformatics, Engineering, Mathematics and computing

## Abstract

Data on body weight, as well as objective measures of body condition and size, are essential for appropriate decision-making on farm level, for example in calculations of nutrient requirements, health monitoring, and breeding-related assessment. Weighing dairy cattle and assessing their body condition are labour-intensive and are therefore often not performed as frequently as desired under practical farm conditions. Although recent research has shown strong potential for computer vision and image analysis in automated estimation of body weight, body condition score, and conformation, many existing workflows still depend on fixed multi-camera or 3D setups that increase hardware and deployment costs. We therefore developed a two-step, smartphone-centred workflow for practical live-weight estimation in dairy cattle. First, a Mask R-CNN segmentation model was trained on 567 manually annotated cow images collected under varied barn conditions and achieved an F1 score of 0.98. Second, body weight was discretized into nine data-driven categories using a Gaussian Mixture Model (BIC-selected), after which the source weight variable was removed to prevent leakage and a leak-safe pipeline (imputation, robust scaling, fold-internal SMOTE, Extra Trees) was trained within the training partition. Primary evaluation used cow-level grouped splitting so that all features based on repeated observations from the same cow remained in a single partition; a PyCaret implementation was used as an independent cross-check. On the 216-image grouped holdout, the tuned Extra Trees model achieved a macro-F1 of 0.936 (95% CI 0.860–0.983) with a 4.2% error rate. These results were obtained on 1080 images collected using the developed camera app and not used for segmentation-model training. The idea is to further streamline the algorithm to allow its downscaling and transition in the form of a smartphone application to be used on-farm as an open-source support tool.

## Introduction

Accurate information on body weight is important for dairy-cattle management, including feeding, health monitoring, treatment decisions, and breeding-related assessment. However, routine weighing of cattle is often difficult to integrate into everyday farm practice. Conventional approaches, such as visual assessment, heart-girth tapes, or fixed weighing systems, are labour-intensive, time-consuming, and may require close physical handling of animals, which can increase stress and create safety risks for both cattle and farm personnel^1^.

These practical limitations have driven growing interest in automated and non-contact alternatives. In Precision Livestock Farming, camera-based systems are increasingly explored as a way to estimate body size, body condition, and body weight from images. Such approaches are attractive because they can reduce manual labour and support more frequent, standardised, and potentially welfare-friendly data collection under farm conditions^1–5^.

At the same time, practical deployment remains a challenge. Many existing imaging workflows depend on dedicated hardware, controlled acquisition geometry, or infrastructure that is not easily transferable to routine herd management. For broader on-farm use, a weight-estimation system must be both accurate and operationally simple, with low hardware burden and a capture procedure that is feasible under ordinary barn conditions.

The present study evaluated whether dairy-cattle body weight can be estimated accurately from smartphone-based imaging using LiDAR-assisted distance normalisation, instance segmentation, and machine-learning analysis.

To position the proposed workflow within the current state of the art, the next section compares conventional and computer-vision-based approaches to cattle weight estimation, with emphasis on imaging modality, deployment challenges, and suitability for everyday farm use.

## Related work and study positioning

### Conventional weight-estimation baselines

Conventional cattle body-weight estimation has traditionally relied on manual body measurements, especially heart girth and body length, because these can be collected with minimal equipment under field conditions. A wide range of equations has been proposed, from simple linear formulae based on heart girth alone to multi-parameter regressions that incorporate body length, age, or alternative anatomical landmarks when girth is less reliable^6–9^. These approaches remain important as practical baselines, but their performance depends on operator skill, animal posture, breed, and the suitability of the selected equation for the target population.

### Computer-vision approaches to cattle weight estimation

Recent computer-vision-based studies have shown that non-contact cattle weight estimation is feasible, but they differ substantially in imaging modality, acquisition geometry, and deployment challenges. One major line of work relies on fixed 3D or depth-camera systems. These approaches can capture rich geometric information and have demonstrated useful predictive performance for body weight, body mass, body condition, and related traits^10–12^. Their main strength is direct access to body shape information. Their main limitation is that they usually require dedicated sensors, extensive calibration, controlled animal presentation, and installation-specific infrastructure.

A second line of work uses 2D RGB imaging combined with image analysis, segmentation, or morphometric feature extraction^2,3,13,14^. These studies show that body-weight information can be recovered from conventional images, especially when the animal outline is well defined and image acquisition is reasonably controlled. Compared with 3D systems, such workflows reduce hardware complexity and may offer better scalability. However, they remain sensitive to framing, pose, occlusion, camera placement, and variation in distance between the animal and the camera.

### Distance control and visual representation in mobile workflows

Recent reviews suggest that the central challenge is no longer whether camera-based cattle weight estimation can work, but how to make it robust enough for routine farm deployment without dedicated infrastructure^1,5^. This challenge becomes more prominent in mobile single-device workflows, where distance variation, viewing angle, inconsistent framing, and barn-specific imaging conditions can substantially affect the extracted features.

Distance control is therefore not a minor technical detail but a core requirement for mobile image-based weight estimation. Previous work on laser-based ranging shows that measurement performance can be influenced by target reflectivity, illumination, atmospheric interference, and angle of incidence^15–17^. In livestock environments, where coat colour, dust, humidity, and lighting cannot be tightly standardised, these factors become especially relevant. A practical mobile workflow must therefore achieve robust scale control under imperfect field conditions while keeping hardware requirements minimal.

The choice of visual representation is also important. Bounding-box detectors provide coarse object extent, whereas segmentation approaches provide pixel-level shape information. For single-image weight estimation, instance segmentation is especially attractive because it preserves silhouette-derived morphometric information, such as projected area, contour geometry, and shape ratios, that are more directly related to body volume proxies than rectangular boxes.

### Study positioning

The present study is positioned within this practical gap. Rather than targeting fixed multi-camera or dedicated 3D systems under highly constrained acquisition conditions, we evaluate a smartphone-centred workflow that combines RGB imaging, LiDAR-assisted distance normalisation, and instance segmentation for dairy-cattle weight estimation under field conditions. The intended contribution is therefore not only predictive performance, but also a more practical acquisition strategy with clearer potential for routine on-farm deployment.

To clarify how the present workflow differs from representative earlier studies, Table 1 compares major methodological families in terms of modality, acquisition setup, output type, practical strength, and deployment limitation.

Based on these practical and methodological gaps, we designed a smartphone-based pipeline that combines controlled image capture, LiDAR-assisted scale normalisation, instance segmentation, and supervised weight classification.

**Table 1:** Comparative overview of representative computer-vision-based approaches to cattle body-weight estimation and their practical deployment trade-offs.

Study	Modality/sensor	Acquisition setup	Primary target output	Reported performance in current manuscript	Main strength	Main deployment limitation
Song et al.^11^	3D vision	Fixed 3D camera in a dedicated passage or weighing setup	Continuous dairy-cattle body-weight estimation	RMSE 41.2 kg; MAPE 5.2%	Strong geometric information and useful predictive accuracy for body weight	Requires stationary hardware, controlled geometry, and dedicated infrastructure
Lee et al.^13^	2D RGB imaging with segmentation	Image-based 2D deep-learning pipeline	Continuous cattle body-weight estimation	MAPE 5.5% with fully supervised segmentation	Shows that segmentation-driven feature extraction from 2D images can support weight estimation	Sensitive to capture conditions and dataset-specific imaging characteristics
Nir et al.^10^	Low-cost 3D computer vision/depth sensing	Dedicated depth-based setup	Heifer height and body mass estimation	Not specified in current manuscript	Rich body-shape information from depth data	Requires dedicated sensors, calibration, and controlled conditions
Spoliansky et al.^12^	Low-cost 3D Kinect-type depth camera	Dedicated 3D sensing setup	Body condition scoring and body-mass-related traits	Not specified in current manuscript	Shows the value of depth-derived morphology for animal assessment	Requires dedicated sensors and controlled acquisition environment
Cominotte et al.^2^	Low-cost 3D Kinect-type depth camera	Dedicated 3D sensing setup	Body weight and average daily gain estimation	RMSE 0.03–11.4 kg for different feeding phases	Supports the feasibility of image-derived morphometrics for production traits	Transferability depends on camera placement, animal presentation, and local context
Ozkaya et al.^14^	2D image analysis	Fixed camera, animals fixed in a chute	Body weight and body-dimension estimation	R^2^ value of 88.7% (body area to body weight)	Demonstrates that conventional 2D imaging can recover weight-related morphometric information	Sensitive to camera placement, presentation, and calibration conditions
Present study	Smartphone RGB imaging with LiDAR-assisted distance normalisation and instance segmentation	Handheld, smartphone-centred field workflow under barn conditions	Ordinal dairy-cattle body weight category estimation	Tuned holdout macro-F1 0.910 (95% CI 0.846–0.983); 9.7% error rate, all adjacent-category errors	Lower hardware requirement, distance normalisation, no need for static setup (potentially lower cost for end-users)	More sensitive to pose, framing, and cross-device variability; requires external validation

Reported performance values are shown only where they are explicitly stated in the manuscript. For several cited studies, the current manuscript uses them for methodological positioning rather than for direct metric-to-metric comparison.

## Methods

### Ethical consideration

Prior to the start of the experiment, the procedures and details of the experiment were evaluated by the Board of Ethical use of Animals in Teaching and Research, Swedish University of Agricultural Sciences, Uppsala, Sweden, and an ethical permit was obtained from the Swedish Board of Agriculture, Uppsala, Sweden ID number: 5.8.18-05598/2021. All procedures were conducted in accordance with the ethical guidelines proposed by the Ethical Committee of the ISAE (International Society of Applied Ethology^18^ and met the ARRIVE guidelines^19^.

### Study location and animals

The data was collected at two locations. At the first location, 248 lactating dairy cows of the breeds Swedish Red (*n* = 148) and Swedish Holstein (*n* = 100) were included for observations over twelve distinct days on nine separate occasions from October 2021 to February 2024. The cows were housed in a free-stall system at the Swedish Livestock Research Centre (Swedish University of Agricultural Sciences (SLU), Uppsala, Sweden). At the second location, 22 cows of the Swedish Red breed were included for observations for one day, to extend the pool of available animals and capture as much individual variability as possible. The cows were housed in a free-stall system at Röbäcksdalen’s Research Facility, SLU, Umeå, Sweden.

### Manual baseline equations and reference measurements

To benchmark conventional morphometric approaches against scale-recorded body weight, several commonly used manual weight-estimation equations were evaluated using measurements collected in Batches 1 and 2. Manual measurements were obtained with a measuring tape (ANIMETER measuring tape, Växa, Jönköping, Sweden) and included chest girth, stomach circumference, back length (diagonal and direct), front and back height, and rump width. Equation outputs were compared with body weights recorded by the automated platform-scale system. This step was used to quantify agreement with manual baselines and to identify body dimensions relevant for subsequent image-based modelling.

### Image acquisition protocol

High-quality images for a well-performing CV model are dependent on a standardized image acquisition protocol. In our study, the protocol specified (1) a stand-off distance that ensured full-body coverage without occlusion by other animals or obstacles, (2) a near-perpendicular camera angle to reduce perspective distortion, and (3) operator positioning that maintained safety and avoided disturbing normal animal behaviour. A commercial Bosch laser distance meter (LDM, Bosch GLM 40 Professional, Robert Bosch Power Tools GmbH, Leinfelden-Echterdingen, Germany) was used during protocol development to monitor stand-off distance; the manufacturer specifies a typical measurement accuracy of ± 1.5 mm for this device.

We evaluated three configurations to quantify practical trade-offs and to guarantee seamless transition to a smartphone-only distance estimation: (a) handheld LDM with a handheld camera/smartphone; (b) tripod-mounted LDM and camera aligned to the cow’s midline and targeting the thoracolumbar region; and (c) a rigid bar (camera plus two LDMs) on a tripod to simultaneously sample distances to the cranial and caudal body, providing a simple approximation of animal depth. Across trials, stand-off distances ranged from 1.44 to 2.72 m; empirically, ~ 2.00–2.05 m yielded the most consistent full-body coverage with minimal occlusion and stable focus and was therefore adopted when barn layout and animal flow permitted. To further stabilise image quality, operators were instructed to avoid strong backlighting, minimise motion during exposure, keep lenses clean, and favour perpendicular incidence to mitigate measurement and future segmentation errors associated with dark, glossy or wet coat patches.

### Mobile application development

**Fig. 1:**
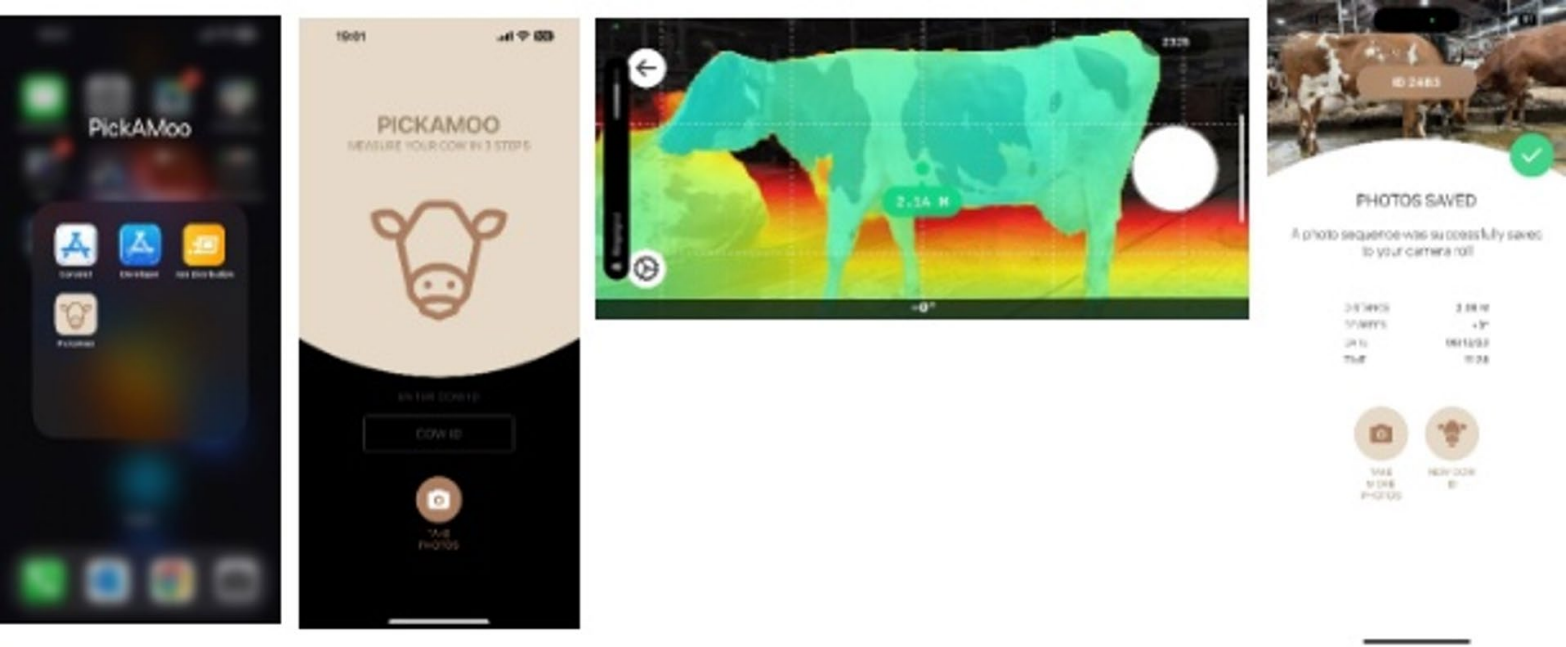
Examples of user interface screenshots from the final version of the PickAMoo app (left to right): (**a**) app selection on the main screen (**b**) welcoming screen where user is requested to enter a cow ID to start the measurement (**c**) LIDAR-based distance and perspective view, where optimal distance for image acquisition is highlighted with green, and changes to red when sub-optimal conditions are detected (**d**) the confirmation of the image being saved, with additional metadata assigned to each photographed animal.

After standardising the image-acquisition protocol, a dedicated mobile application was developed to integrate distance estimation, image capture, and future weight-estimation functionality into a single workflow. Development and interface design were contracted to a digital interaction agency with experience in app prototyping and user-interface engineering. Apple iPhone Pro and Pro Max devices (iPhone 12 Pro and newer; Apple Inc., Cupertino, CA, USA) were selected because they provide application-level access to the LiDAR sensor, which was required for stand-off estimation and scale control during image capture. The development process followed four coordinated workstreams: (1) a camera module for controlled exposure, focus, and resolution, (2) a distance module for real-time LiDAR-based guidance within a target operating window, (3) classifier integration for handling computer-vision outputs and feature computation, and (4) a user-interface and data layer for animal selection, capture confirmation, and record keeping. Iterative weekly builds were delivered for field testing and feedback, resulting in seven app versions. The final data-acquisition app, PickAMoo (Fig. 1), included a burst mode that captured five consecutive images per animal, LiDAR-based distance estimation with depth-map preview, and a gyroscope-based tilt indicator to support near-perpendicular framing.

### LIDAR/LDM distance measurement consistency

**Fig. 2:**
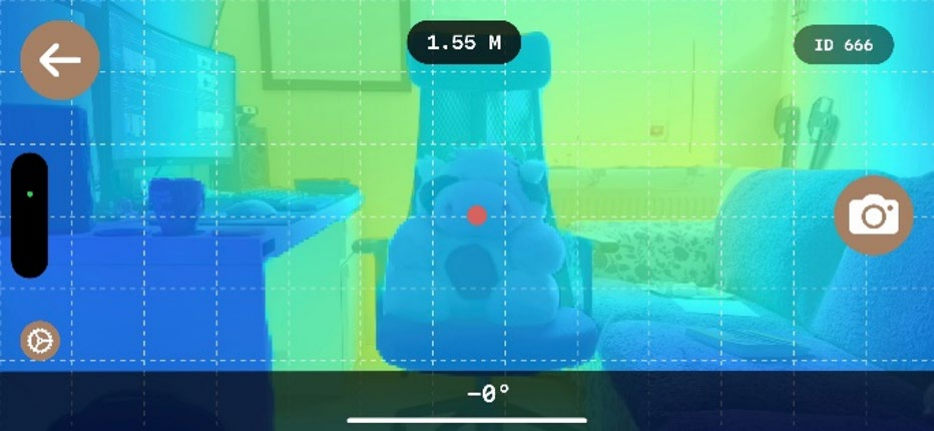
Experimental setup for worst-case reflectivity testing. A toy cow with contrasting black and white fur was used as a surrogate object to evaluate LiDAR performance under challenging surface conditions. Dark fur patches acted as low-reflectivity regions, allowing assessment of the system’s ability to maintain distance accuracy on non-uniform targets.

A central methodological challenge was to ensure that images were acquired at a sufficiently consistent distance and angle to support reliable scale normalisation. To address this, the agreement and operational consistency of the external laser-distance meter and the smartphone-based LiDAR interface were evaluated under typical barn conditions and under a deliberately difficult reflectance scenario. These checks were used to refine capture guidance, confirm a practical working-distance window, and identify situations in which dark, low-reflectance regions could transiently destabilise depth readings. The purpose of this step was not to treat smartphone LiDAR as a millimetric ground-truth instrument, but to ensure sufficiently robust capture conditions for downstream image analysis. During early app testing, occasional deviations in distance estimation were observed. A worst-case reflectance test was therefore performed using a toy cow with highly contrasting black and white fur (Fig. 2). The darkest fur regions produced the largest measurement instability, confirming that low-reflectance surfaces could compromise distance estimation under challenging conditions. The resulting information was used to recalibrate the LiDAR sensor of the test device (iPhone 14 Pro Max) and to refine the image-acquisition protocol. During the final round of data collection, 1300 images were captured at consistent distances without notable measurement errors.

### Data collection and image annotations

Data (images, BW and manual measures) were gathered on 12 separate days spread over three and a half years, with different animals included at each session to capture a representative cross-section of the herd (Table 2). To avoid within-day duplication, no individual cow was weighed or photographed more than once on the same day. Across nine data-collection rounds, the final dataset comprised 2847 images suitable for analysis. Imaging was performed with an Olympus µ−700 digital camera and a Motorola smartphone during Batches 1 to 6, and with an iPhone 14 Pro Max running the experimental PickAMoo application during Batches 7 to 9. The dataset comprised 248 unique cows photographed across multiple sessions under varying imaging conditions. Manual body measurements (e.g., heart girth, body length, height, rump width) were collected for Batches 1–2 and paired with ground-truth body weights from the barn’s automated scales (Batches 1–9), providing a reference set to benchmark manual equations and to anchor subsequent image-based modelling. Together, these multi-device, multi-session data constituted a heterogeneous basis for developing and stress-testing our CV and machine learning pipelines.

**Table 2:** Overview of the data collection occasions (Batches) with number of images, animals for each occasion and whether manual measurements complementary to automatic weighing were taken (*Updated protocol for distance measurement (two reference points instead of one) **Data collected using a PickAMoo app with LIDAR-functionality for distance estimation).

Batch #	Date	# of images	# of animals photographed	Manual measurements
1a	May 2021	256	34	Yes
1b		258	28	Yes
2	July 2021	55	22	Yes
3a	October and November 2021	232	92	No
3b		166	54	No
3c		100	52	No
4*	October 2022	14	5	No
5*	November 2022	305	37	No
6*	November 2022	125	25	No
7*	February 2023	110	17	No
8**	May 2023	104	26	No
9**	December 2023	1122	145	No
Total		2847	537	

**Fig. 3:**
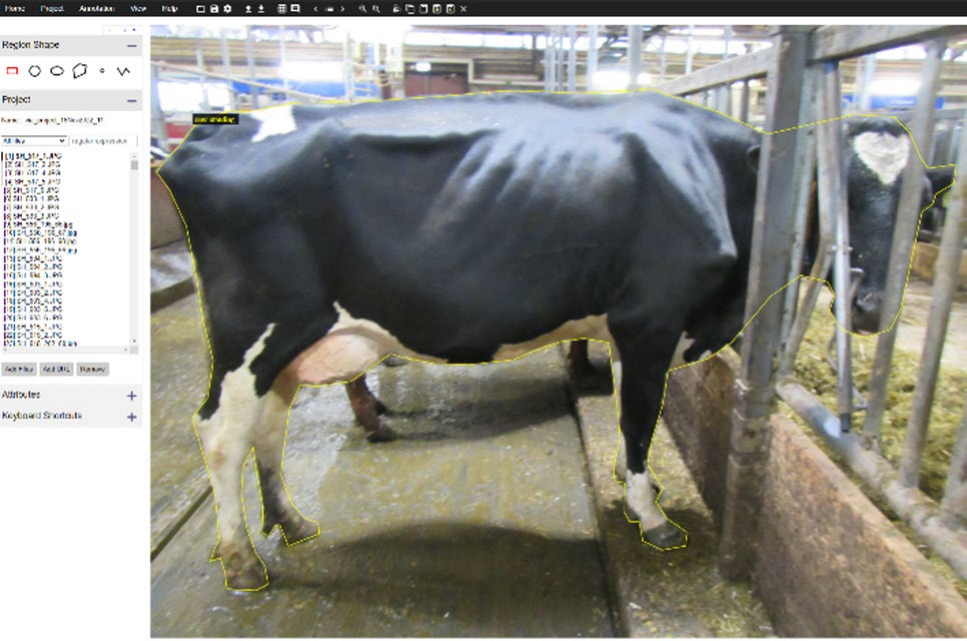
Example image of a cow in one of the typical barn contexts. Yellow line represents a manually drawn polygon which served as the Ground Truth for training the segmentation model.

To create quality backbone for image segmentation model, 567 images were manually annotated using the VIA Image Annotator (Visual Geometry Group, University of Oxford, Oxford, UK). Annotators draw precise polygons around each focal cow to produce instance masks suitable for training Mask R-CNN model (Fig. 3). Annotation guidelines prioritised full-body contours when visible and resolved partial occlusions by following the visible outline only; images with severe occlusion or motion blur were excluded from the gold-standard set.

These masks formed the basis for training and validating our instance-segmentation models and for deriving silhouette-based features (e.g., projected area, aspect ratios and contour descriptors) used in weight classification.

### Development of object detection/segmentation model

For segmentation-model development, the manually annotated reference dataset (*n* = 567) was split at cow level into training, validation, and test subsets so that no cow contributed images to more than one partition. The validation subset was used for model selection and early stopping, whereas the test subset was reserved for final reporting. The split comprised 75% training images (*n* = 425), 15% validation images (*n* = 85), and 10% test images (*n* = 57). Two segmentation architectures were compared: U-Net and Mask R-CNN. In preliminary experiments, U-Net produced unstable silhouettes in cluttered and partially occluded barn images, whereas Mask R-CNN more consistently separated the focal cow from nearby animals and barn structures. Model development therefore focused on Mask R-CNN.

Four pretrained backbones were evaluated within the Mask R-CNN framework: EfficientNet-B7, MobileNetV3, ResNet-101, and DenseNet-201. Common training settings were used across backbones: two images per GPU, 50 epochs, 1000 steps per epoch, 50 validation steps per epoch, an initial learning rate of 0.001, momentum of 0.9, weight decay of 0.0001, and two classes (cow and background). Early stopping was applied to reduce overfitting. Models were trained and evaluated on a workstation equipped with an AMD Ryzen 5950X CPU, 128 GB DDR4-3600 RAM, and an NVIDIA RTX 4080 GPU with 16 GB VRAM. In addition to quantitative evaluation, each model was applied to a separate random set of 150 images sampled from the full dataset for qualitative stress testing under varied barn conditions. These images were not used for model selection or headline performance reporting. ResNet-101 produced the most reliable instance masks on held-out and previously unseen images while maintaining acceptable inference speed and was therefore selected for downstream feature extraction.

### Weight classification model

**Fig. 4:**
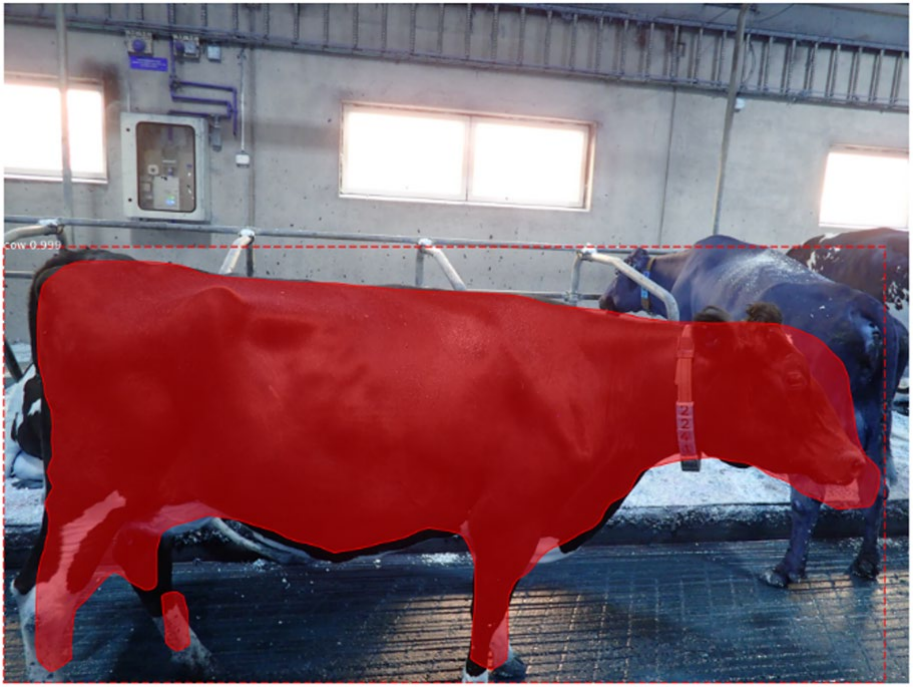
Results of an object detection by Mask R-CNN (rectangular bounding box around the detected object, here cow) and a segmentation mask of said object.

For each detected cow, Mask R-CNN produced both a bounding box and a per-pixel segmentation mask (Fig. 4). From these outputs, a compact set of silhouette-based features was extracted, including bounding-box width, height, and area; mask area; extent; convex-hull area; elongation; contour length; and moment-based descriptors. Binary masks were cleaned using light morphological operations, including hole filling and removal of small, connected components, to improve measurement stability across poses and backgrounds.

Because all pixel-derived features depend on stand-off distance, a per-image scale normalisation was applied using the recorded distance value. A pixels-to-millimetres factor was computed and all length- and area-based features were re-expressed relative to a common 2.00 m reference distance. To improve generalisability, a single external scalar covariate reflecting body girth was also included. When manual measurements were unavailable, the cohort-average heart girth of approximately 200 cm was used as a constant prior.

The final dataset for weight-classification modelling contained 1080 entries derived from 1080 images in Batch 9, each matched to an exact body weight recorded by the automatic scale system. Because farm decision-making often relies on weight bands rather than exact kilograms, the continuous weight variable was transformed into a categorical target using a one-dimensional Gaussian Mixture Model. The number of mixture components was selected by minimising the Bayesian Information Criterion over K = 3 to 10, and clusters containing fewer than 25 animals were merged with the nearest cluster in mean weight. The resulting ordered target variable was denoted AutoWeightCategory.

Supervised classification was implemented in Python. The primary workflow used scikit-learn and imbalanced-learn, while PyCaret was used as an independent implementation check on the same training partition. A single 80:20 train-holdout split was created at cow level, with the aim of preserving the derived weight-category distribution as closely as possible. For each capture event, multiple images of the same cow were segmented individually, image-derived, weight-related features were calculated for each image, and these features were then aggregated to a single capture-level observation before model development. This burst-level aggregation was specifically introduced to reduce within-event variability caused by pose, minor segmentation differences, and momentary image-acquisition noise.

The holdout set remained untouched until final evaluation. All model development was performed within an end-to-end pipeline that included median imputation, robust scaling, removal of zero-variance features, fold-internal SMOTE for class-imbalance handling, and an Extra Trees classifier with 500 trees, balanced class weights, parallel execution, and a fixed seed.

Model selection was performed within the training partition using cross-validation that respected cow-level grouping, so that no cow contributed images to more than one fold. Macro-F1 was specified a priori as the primary metric, while weighted-F1 and accuracy were retained as secondary summaries. After cross-validation, the final pipeline was refitted on the full training partition and evaluated once on the untouched grouped holdout set. Because the target classes were ordinal, prediction errors were also characterised by absolute class distance, including the number of adjacent and non-adjacent misclassifications, as well as mean, median, and maximum distance. Uncertainty on holdout macro-F1 was estimated using bootstrap resampling with 2000 replicates. Predicted probabilities from the scikit-learn pipeline were further assessed using the multiclass Brier score, expected calibration error, and a reliability diagram. For the independent confirmation step, PyCaret was run with internal resampling disabled, and a class-weighted Extra Trees model was tuned, finalised, and evaluated on the same grouped holdout set.

## Results and discussion

### Performance of manual weight-estimation equations

The manual weight-estimation equations showed substantial variation in performance across breeds and predictor sets (Table 3). In the mixed-breed dataset, the equation combining heart girth, body length, and age produced the best overall fit, with R² = 0.89 and a mean absolute percentage error (MAPE) of 4.47%. Models based on heart girth alone performed less well, indicating that the inclusion of additional body measurements improved predictive accuracy.

Breed-specific analyses showed clear differences. In Swedish Red cows, all evaluated equations performed less well, with R² values of 0.69 to 0.72 and MAPE values of 13.34 to 14.12%. In Swedish Holstein cows, the same equation family performed markedly better, with the best model reaching R² = 0.93 and MAPE = 3.55%. All regressions were highly significant (*p* < 2.2 × 10⁻¹⁶). These results show that conventional morphometric equations remain useful as baseline methods, but their performance depends strongly on breed and predictor choice.

In addition, cows weighing more than 800 kg showed higher prediction error than lighter cows. In the present dataset, these heavier animals were predominantly Swedish Red, which may partly explain the reduced fit of the equations in that subgroup. The lower body weight to heart-girth correlation observed in Swedish Red cows further supports the presence of breed-specific conformational effects on manual weight estimation.

**Table 3:** Comparison of equations for body weight estimation in cattle and their estimated accuracy when applied to Swedish Red and Swedish Holstein breeds (BW – Body Weight, HG – Heart Girth, BL – Body Length).

Breed	Formula	*R*²	*p*-Value	Mean Absolute Percentage Error (MAPE)
Mixed Breeds (SR and SH)	HG*7.3827–878.3134	0.85	< 2.2e-16	5.42%
	HG*6.2570 + BL*2.3311–1035.94	0.87	< 2.2e-16	4 0.89%
	HG*5.4143 + BL*2.4066 + AGE*11.2416–911.4412	0.89	< 2.2e-16	4.47%
Swedish Red (SR)	BW = HG*7.1984–851.8974	0.69	< 2.2e-16	14.12%
	BW = HG*6.1404 + BL*2.0569–975.8509	0.71	< 2.2e-16	13.67%
	BW = HG*5.4592 + BL*2.0203 + AGE*11.8829–869.3609	0.72	< 2.2e-16	13.34%
Swedish Holstein (SH)	HG*7.455–891.57	0.90	< 2.2e-16	4.07%
	6.7215 + BL* 1.9233–1065.8118	0.91	< 2.2e-16	3.59%
	HG*5.7649 + BL*2.1257 + AGE*8.6201–928.2627	0.93	< 2.2e-16	3.55%

### Segmentation model performance

The two segmentation architectures differed substantially in performance on barn images. U-Net underperformed on cluttered and partially occluded images, with an F1 score of 0.56. In contrast, Mask R-CNN consistently produced stable and biologically plausible cow silhouettes and achieved a final F1 score of 0.98. Among the evaluated Mask R-CNN backbones, ResNet-101 yielded the most reliable masks on held-out images and on additional unseen images used for qualitative stress testing.

**Fig. 5:**
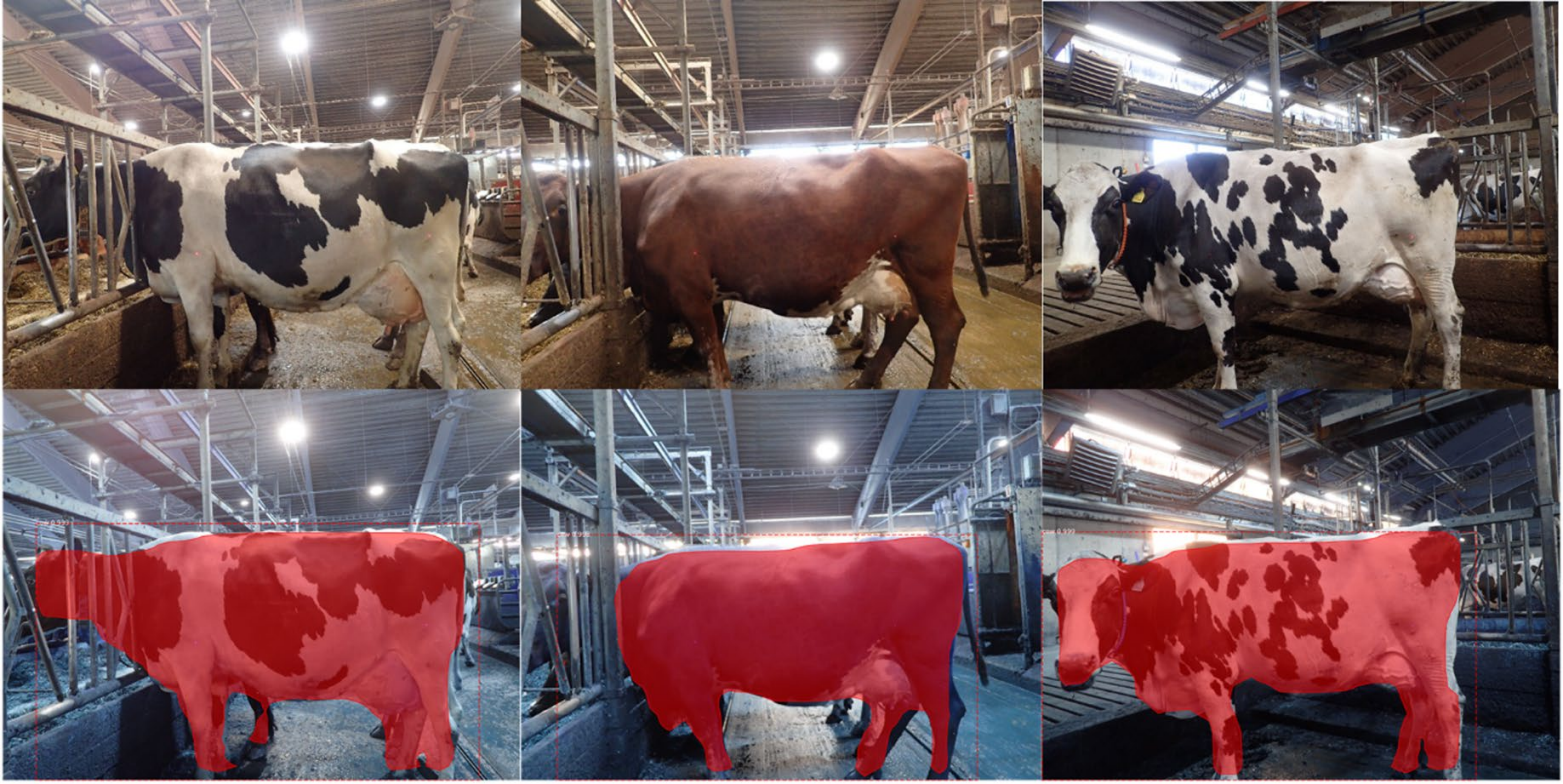
Model detection/segmentation real-world variability.

The main segmentation errors involved variable inclusion of extremities and partially obscured body parts, including the head, tail, ears, and distal limbs (Fig. 5). These inconsistencies altered final mask size and had the potential to affect downstream feature extraction. However, the combination of burst-image capture, shape-aware feature extraction, and the inclusion of a low-variance body-girth prior reduced the impact of such local mask variation on the final weight-classification step.

### Image-based weight classification model performance

The final classification dataset consisted of 1080 images from Batch 9, each linked to exact body weight recorded by the automatic scale system. Continuous body weight was transformed into nine ordered categories, with a minimum class size of 25 animals. The primary evaluation used cow-level grouped splitting so that all images belonging to the same cow, were retained within a single partition. The grouped holdout contained 216 images and served as the basis for the main performance claim.

On the grouped holdout set (*n* = 216), the primary scikit-learn pipeline achieved a macro-F1 of 0.910 (95% bootstrap CI: 0.846–0.953), a weighted-F1 of 0.904, and an accuracy of 0.903. The overall error rate was 9.7%. Most errors were adjacent-category mistakes (7.4%), whereas non-adjacent errors were less frequent (2.3%). The mean absolute class distance was 0.130, the median was 0, and the maximum was 3 categories. Probability calibration analysis gave a multiclass Brier score of 0.2114 and an expected calibration error of 0.2112, indicating moderate overconfidence at higher predicted probabilities.

The independently tuned PyCaret Extra Trees model improved grouped-holdout performance. Macro-F1 increased to 0.936 (95% bootstrap CI: 0.860–0.983), while weighted-F1 and accuracy reached 0.957 and 0.958, respectively. The overall error rate was 4.2%. These results indicate that the final workflow retained strong performance when evaluation was constrained at cow level.

## Discussion

### Main findings

This study showed that dairy-cattle body weight can be estimated accurately from smartphone-based imaging when two technical constraints are addressed together: consistent scale normalisation during image capture and robust extraction of shape-aware visual features. The final workflow combined LiDAR-assisted stand-off control, Mask R-CNN instance segmentation, and feature-based machine learning, and it retained strong performance when the primary evaluation was performed with cow-level grouped splitting. On the grouped holdout, the tuned model reached a macro-F1 of 0.936 with a low overall error rate.

The manual baseline analyses also provided useful context. Conventional equations performed reasonably well in the mixed-breed sample and very well in Swedish Holstein cows, but much less well in Swedish Red cows. This confirms that low-cost manual approaches remain relevant as practical baselines but also highlights their sensitivity to breed-related conformational differences. In contrast, the image-based workflow was designed to recover shape information directly from images and to reduce dependence on fixed formulae derived from specific body-measurement relationships.

### Why the workflow performed well

Several design choices likely contributed to the observed performance. First, scale variation was addressed explicitly through LiDAR-assisted distance control and per-image normalisation to a common 2.00 m reference. This is important because variable stand-off distance is a major source of instability in single-image morphometric analysis. Second, instance segmentation was used instead of coarse bounding-box detection, which allowed extraction of silhouette-derived descriptors more closely related to body volume proxies. Third, all preprocessing, resampling, and model fitting steps were confined within cross-validation folds, and final reporting was based on a grouped holdout in which repeated observations from the same cow were not allowed to cross partitions. This reduced the risk of leakage and made the reported performance more trustworthy.

The error structure also supports the biological plausibility of the model. In the primary scikit-learn evaluation, most residual errors occurred between neighbouring weight categories, which is expected when thresholds are imposed on an underlying continuous trait. This pattern suggests that the classifier captured meaningful variation in body mass rather than spurious image artifacts. The higher but broadly consistent performance obtained with the independent PyCaret implementation further strengthens confidence that the findings did not depend on one specific software pipeline.

### Practical relevance

A central contribution of the study is practical rather than purely algorithmic. Many earlier cattle weight-estimation systems rely on fixed 3D setups, multiple cameras, or controlled passage geometry. Such systems can perform well, but they impose infrastructure demands that limit routine on-farm use. In contrast, the present workflow was built around a handheld smartphone-based acquisition strategy with integrated distance guidance and a simple capture procedure. This substantially lowers the hardware requirements and improves the realism of everyday deployment.

The workflow may therefore be useful in settings where approximate but reliable weight-band assignment is sufficient for management decisions. Examples include rapid triage for feeding adjustments, treatment support, and longitudinal monitoring of body-weight trajectories with limited animal handling. The ability to capture repeated, standardised measurements using a mobile device also creates opportunities for building shared data streams that could support advisory services, herd monitoring, and future decision-support tools.

### Limitations

The study should still be interpreted as a proof of concept. The dataset was heterogeneous and collected across multiple sessions and devices, but it remained geographically limited to two Swedish research herds and was dominated by two breeds. The weaker performance of manual equations in Swedish Red cows, especially at higher weights, suggests that breed-related differences in body conformation may also affect broader model transferability. External validation on additional breeds, production systems, and farm environments is therefore needed before wider deployment.

Further limitations concern both modelling and deployment. The final task was ordinal, but a standard multi-class framework was used rather than an explicitly ordinal objective. Reflectivity-related edge cases can still affect LiDAR-derived depth estimation in principle, even though the final field round did not show notable practical failures after recalibration. In addition, the current Mask R-CNN configuration is not yet optimised for on-device inference. Compression, pruning, or quantisation will be needed for fully mobile deployment, and these steps must be evaluated carefully to ensure that accuracy remains stable across subgroups and imaging conditions.

### Future directions

Three development paths appear most important. First, the workflow should be tested prospectively across a broader range of breeds, housing systems, seasons, and countries, with external validation against calibrated scales and explicit subgroup reporting. Second, capture and modelling could be more tightly integrated by enforcing silhouette-quality checks during acquisition and by fusing information across repeated, short burst sequences rather than relying on an aggregated single frames alone. Third, edge deployment should be pursued through model compression and on-device inference, combined with secure and controlled updating procedures for recalibration over time.

Taken together, the present results support the feasibility of smartphone-based, non-contact weight estimation in dairy cattle under realistic barn conditions. The workflow does not yet replace calibrated weighing systems, but it demonstrates that a lower-tier hardware and operationally realistic alternative can achieve strong predictive performance while preserving practical usability.

## Conclusions

This study evaluated whether dairy-cattle body weight can be estimated accurately from smartphone-based imaging using LiDAR-assisted distance normalisation, instance segmentation, and machine-learning analysis.

The results demonstrated that accurate, non-contact estimation of dairy-cattle body weight is achievable when two long-standing imaging bottlenecks are addressed jointly: (1) reliable scale normalisation at capture time and (2) segmentation quality sufficient to extract shape-aware features that correlate with mass. By coupling iPhone-class LiDAR for stand-off control with a Mask R-CNN (ResNet-101) instance-segmentation pipeline and a lightweight, feature-based estimator, robust weight estimation under real-world barn conditions was possible. Importantly, this conclusion remained supported when the primary evaluation was performed with cow-level grouped splitting.

## Data Availability

The data and custom code that support this study are available from the corresponding author on reasonable request. Public deposition is temporarily restricted due to a pending patent investigation. We will release the data and code in a public repository once the patent review is complete. During peer review we will provide editors and reviewers with all necessary data and code on request.
